# Functional Expression and Characterization of the Recombinant *N*-Acetyl-Glucosamine/*N*-Acetyl-Galactosamine-Specific Marine Algal Lectin BPL3

**DOI:** 10.3390/md16010013

**Published:** 2018-01-05

**Authors:** Hyun-Ju Hwang, Jin-Woo Han, Gwang Hoon Kim, Jong Won Han

**Affiliations:** 1 Department of Genetic Resources Research, National Marine Biodiversity Institute of Korea, Seocheon 33662, Korea; hjhwang@mabik.re.kr (H.-J.H.); hiclow@mabik.re.kr (J.-W.H.); 2 Department of Biology, Kongju National University, Kongju 32588, Korea

**Keywords:** *Bryopsis plumosa*, BPL3, lectin, hemagglutinin, recombinant, tandem repeat, GlcNAc, GalNAc

## Abstract

Lectins, characterized by their carbohydrate-binding ability, have extensive practical applications. However, their industrial use is limited due to impurity. Thus, quality-controlled production of recombinant lectin is necessary. In this study, the algal lectin BPL3 (*Bryopsis plumosa lectin* 3) was successfully produced using a bacterial expression system, BL21(DE3), with an artificial repeated structure (dimeric construct). Recombinant dimeric BPL3 (rD2BPL3) was confirmed by LC-MS/MS spectrometry. Expression efficiency was greater for the construct with the repeat structure (rD2BPL3) than the monomeric form (rD1BPL3). Optimal conditions for expression were 1 mM IPTG at 20 °C. Recombinant lectin was purified under denaturing conditions and refolded by the flash dilution method. Recombinant BPL3 was solubilized in 1× PBS containing 2 M urea. rD2BPL3 showed strong hemagglutination activity using human erythrocyte. rD2BPL3 had a similar sugar specificity to that of the native protein, i.e., to *N*-acetyl-glucosamine (GlcNAc) and *N*-acetyl-galactosamine (GalNAc). Glycan array results showed that recombinant BPL3 and native BPL3 exhibited different binding properties. Both showed weak binding activity to α-Man-Sp. Native BPL3 showed strong binding specificity to the alpha conformation of amino sugars, and rD2BPL3 had binding activity to the beta conformation. The process developed in this study was suitable for the quality-controlled large-scale production of recombinant lectins.

## 1. Introduction

Lectins are well-known carbohydrate-binding proteins able to agglutinate cells by glyco-conjugation; they have many medical and scientific applications [1]. For example, lectins are a potential diagnostic molecule for carbohydrate profiling on cell surfaces [2] and can be used for the identification of glycoproteins [3]. Fluorescently labelled lectins have been used for the visualization of polysaccharides in biofilms of *Pseudomonas aeruginosa* [4]. Lectin affinity chromatography has become a common method for the isolation of glycoproteins from cell extracts [5]. Recently, the application of silver nanoparticles with C-type lectin as a recognition ligand has been suggested for bacterial detection [6]. Lectin histochemistry has also be used for the diagnosis of *Sida carpinifolia* (Malvaceae) poisoning in sheep [7]. An efficient strategy for the systematic production of recombinant lectins for use in microarray technology has also been described [8].

The utility of GalNAc-specific lectins has been reported by several research groups. Gal/GalNAc-specific lectin is a vaccine candidate for amoebiasis and a focus of immunogenicity studies [9]. *Wisteria floribunda* agglutinin (WFA), a GalNAc-specific lectin, shows promise for cancer biomarker detection, with disaccharide LacdiNAc (β-d-GalNAc-[1→4]-d-GlcNAc) recognition properties [10].

To date, approximately 800 algal species have been screened and approximately 60% of these taxa show lectin activity [11]. However, only a few algal lectins (about 50 lectins from marine algae) have been isolated and characterized owing to interfering substances, such as polyphenols, in algae. Insufficient algal biomass is another barrier to the application and commercialization of algal lectin [11]. To overcome these limitations, recombinant techniques are a potentially useful tool for the production and biochemical characterization of active algal lectins.

BPL3 is a previously isolated GlcNAc/GalNAc-specific lectin [12]. This protein and other *B. plumosa*-derived lectins (Bryohealin and BPL4) have important functions in the wound healing process of *B. plumosa* during protoplast regeneration from mechanically damaged cells [12,13]. BPL3 is similar to H-type lectin, which is produced by invertebrates, and not by plants [12]. Based on comparative sequence analyses and the conservation of active sites between BPL3 and the H lectin group, BPL3 was suggested as a research tool in various fields within biochemical and medical sciences [12]. These sequence analyses also suggest that BPL3 is an example of parallel evolution across species boundaries. Despite its overall importance, its biochemical properties, including active sites, are still unclear owing to inability to produce high quantities of pure protein. The production of recombinant BPL3 has not been reported.

Most plants and algae have a heterogeneous mixture of lectin isoforms with diverse biological activities; therefore, a lectin isolated from natural sources is typically not preferred for medical applications [14,15]. In addition, the inability to obtain large amounts of lectins from natural sources is a major hurdle for medical uses. The production of lectins by recombinant techniques was a major break-through, but production of the active form is difficult using bacterial expression systems [16]. Many plant lectins have a dimeric or multimeric structure with homologous subunits exhibiting covalent or non-covalent interactions, and this is demanding in bacterial expression systems. It requires the precise optimization of hydrogen or salt concentrations, which may be un-controllable and difficult to reproduce.

Tandem repeat domain structures have been reported in native lectin from *Silurus asotus* eggs [17], mannose-binding lectin from *Boodlea coacta* [18], and lectin from *Aglaothamnion callophyllidicola* [19]. For example, rhodobindin, a lectin produced from the red alga *A. callophyllidicola* involved in the cell–cell recognition process during sexual reproduction [19], consists of an internal tandem repeat structure with at least eight domains. The tandem repeat structure contributes to the production of the active protein and influences recombinant expression [14].

Based on these previous results for rhodobindin, we predicted that the construction of internal tandem repeat domains may be useful for the production of active lectin. In this study, active recombinant BPL3 was produced with artificial internal tandem repeat domains and its biochemical properties were characterized. The potential applications of this recombinant lectin for biochemical and medical research are discussed.

## 2. Results

### 2.1. Cloning of rBPL3

**Figure 1:**
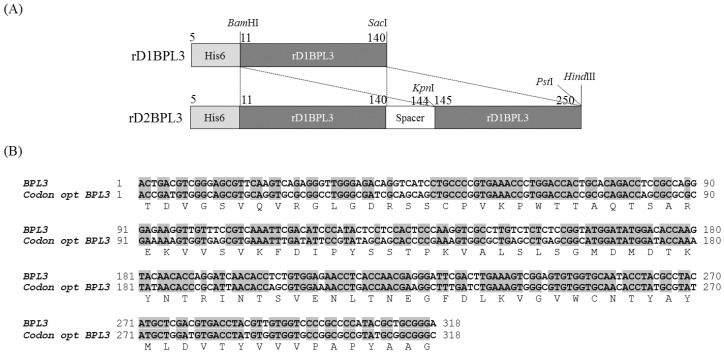
Construction of rD1BPL3 and rD2BPL3. (**A**) Monomeric and dimeric forms of rBPL3; (**B**) Codon-optimized BPL3.

**Figure 2:**
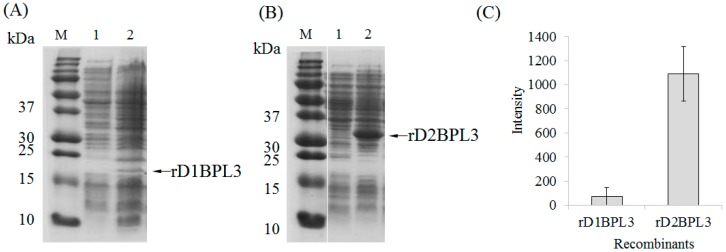
Expression efficiency of rD1BPL3 and rD2BPL3 according to repeated sequences. (**A**) rD1BPL3 expressed in BL21(DE3), (**B**) rD2BPL3 expressed in BL21(DE3). M, Molecular weight marker; Lane 1, un-induced lysate; Lane 2, IPTG-induced lysates. Arrows indicate target proteins. (**C**) Comparison of target protein intensity depending on the repeat sequence array. Error bars indicate standard deviation. *n* = 5.

*BPL3* cDNA was codon-optimized to avoid codon mismatches between marine green alga and bacterial tRNA (Figure 1). The expression efficiency of un-optimized cDNA was inadequate in normal conditions (37 °C, overnight incubation; data not shown), but codon-optimized *BPL3* cDNA was expressed (Figure 2). To determine the effect of a repeated sequence array of homologous domains on hemagglutination activity and expression efficiency, monomeric (rD1BPL3) and dimeric (rD2BPL3) sequences were constructed. The expression efficiency of rD2BPL3, i.e., the dimeric form, was more than 10-fold greater than that of the monomeric form of rBPL3, rD1BPL3 (Figure 2).

### 2.2. Selection of an Expression Host and Optimization of Conditions

BL21(DE3) was chosen as an expression host for rD2BPL3; other bacterial hosts showed similar expression patterns to that of BL21(DE3) at 37 °C (Supplementary Figure S1).

**Figure 3:**
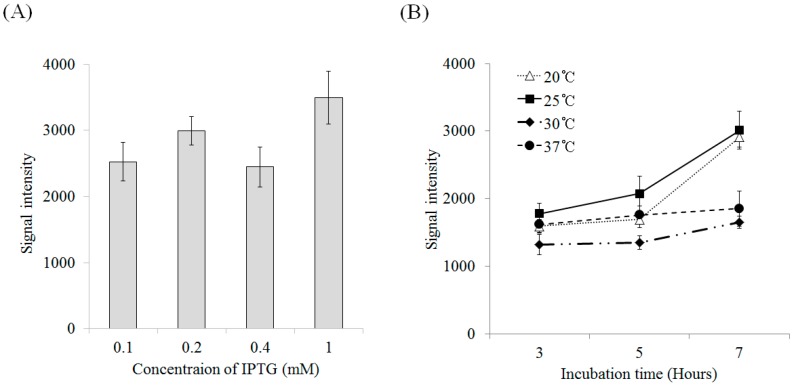
Expression efficiency of recombinant lectin in various conditions. (**A**) Induction efficiency for various concentrations of IPTG (at 37 °C, OD_600_ = 0.4–0.6, overnight); (**B**) The effects of temperature (20–37 °C) and incubation time (3–7 h) on the induction of recombinant protein (OD_600_ = 0.4–0.6, 0.4 mM IPTG).

The expression efficiency of recombinant lectin was not highly affected by IPTG (isopropyl-β-d-thiogalactopyranoside) at various concentrations at 37 °C. The protein expression level with 1 mM IPTG was about 1.5-fold higher than those for the other conditions, but all expression levels were within the margin of error (Figure 3A). Temperature was an essential factor for protein induction. The expression of rD2BPL3 was highest at 25 °C and lowest at 30 °C after 7 h of induction. Increased induction durations influenced the production of rD2BPL3 at 20 °C and 25 °C, but not at normal temperatures (i.e., 30 °C and 37 °C) (Figure 3B). All expression hosts produced inclusion bodies in the insoluble form in the tested conditions (Supplementary Figure S1).

### 2.3. Purification of Recombinant Lectins

**Figure 4:**
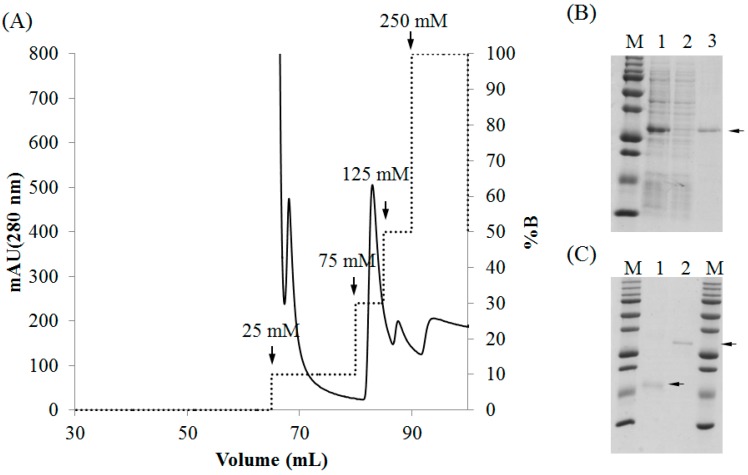
Purification of recombinant lectins by Ni-NTA agarose chromatography. (**A**) Chromatogram showing protein elution from the column; (**B**,**C**) SDS-PAGE; (**B**) Purification of rD2BPL3, M, molecular weight marker; lane 1, crude extract; lane 2, flow-through fraction; Lane 3, purified rD2BPL3; (**C**) Purified recombinant lectins; M, Molecular weight marker; lane 1, rD1BPL3; lane 2, rD2BPL3.

Both rD1BPL3 and rD2BPL3 were solubilized in denaturing conditions (8 M urea in PBS), but not in PBS alone (lacking urea). Ni-NTA agarose was used for the purification of recombinant lectins. Most recombinant lectins were bound to the affinity matrix. Recombinant lectin was eluted by a stepwise gradient of 75 mM, 125 mM, and 250 mM imidazole (Figure 4A). A single band was observed from the eluted fraction for each recombinant (Figure 4B,C). Approximately 3 mg of rD2BPL3 was obtained from 1 L bacterial culture, while rD1BPL3 was obtained in sufficient quantities for further analysis despite the presence of a purified band in Figure 4C. The amino acid sequence of the recombinant protein was confirmed by LC-MS/MS spectrometry (Supplementary Figure S2).

**Figure 5:**
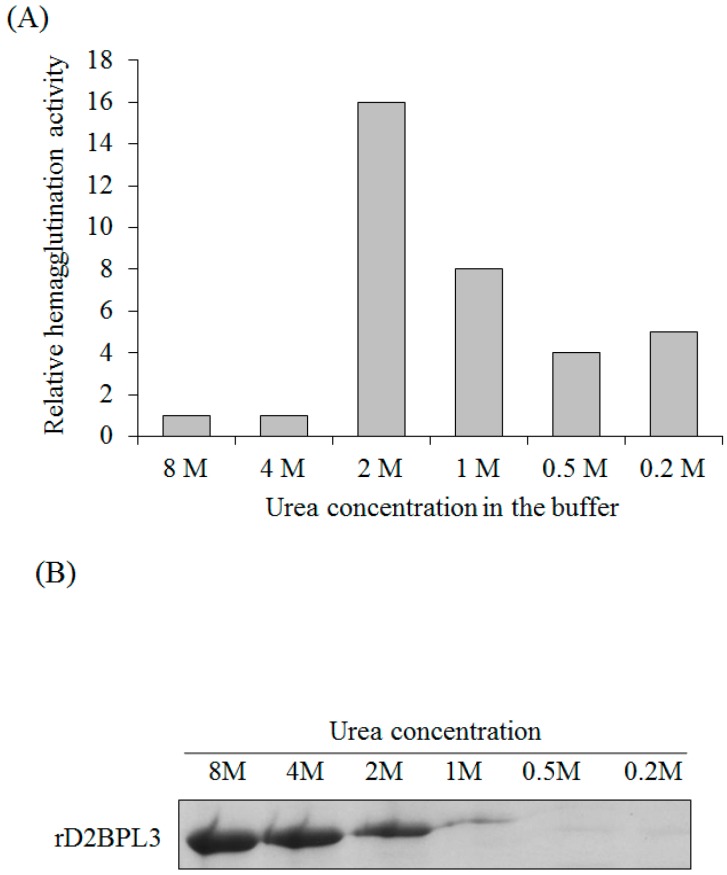
Refolding of rD2BPL3 inclusion bodies (IBs) by the flash refolding method. (**A**) Relative hemagglutination activity of rD2BPL3 at various concentration of a denaturant, urea (0.2–8 M); (**B**) SDS-PAGE, solubility of rD2BPL3 using various concentrations of urea. After flash refolding, insoluble components were removed by centrifugation and the supernatant was subjected to SDS-PAGE. See Materials and Methods section.

The flash dilution method was effective for rD2BPL3 refolding. The dialysis method accelerated the production of inclusion bodies; the protein formed inclusion bodies, when the dialysis buffer contained less than 4 M urea (data not shown). Purified proteins were diluted in 0.2 to 4 M urea in PBS buffer. The hemagglutination activity when the protein was diluted in 2 M urea was 16-fold greater than that in the 8 M urea condition (Figure 5A). The protein was precipitated and a loss of activity was observed at less than 2 M urea (Figure 5). The optimal concentration of urea for refolding was 2 M (Figure 5).

### 2.4. Carbohydrate Specificity and Heat-Stability of Recombinant Lectin

**Figure 6:**
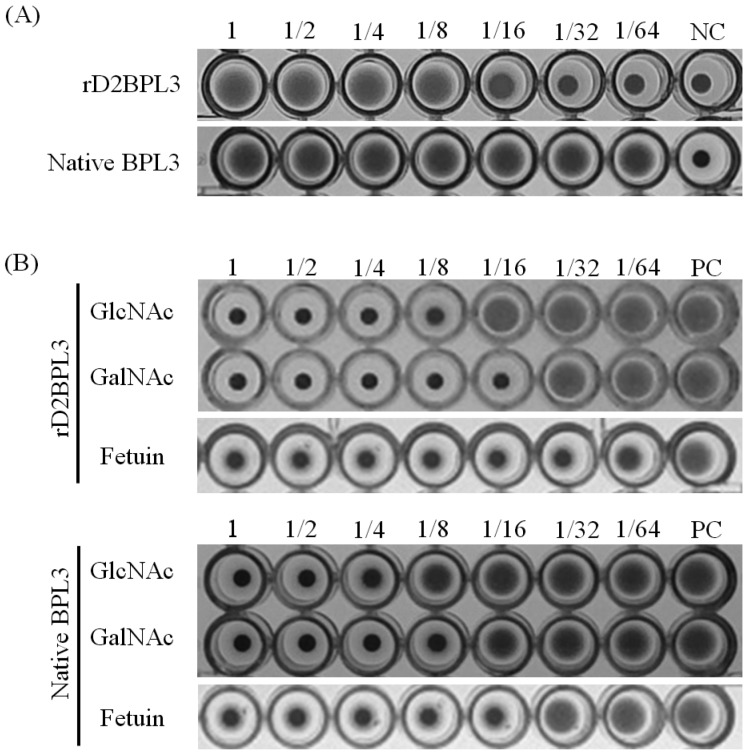
Hemagglutination activity and inhibition of rD2BPL3 (**A**) Hemagglutination activity of rD2BPL3 (100 μg/mL) and native BPL3 (50 μg/mL); A serial twofold dilution was obtained (left to right); (**B**) Inhibition test of rD2BPL3 (25 μg/mL) and native BPL3 (1.5 μg/mL); NC, negative control; PC, positive control.

The recombinant lectin rD2BPL3 showed agglutination activity using human blood cells. The minimum concentration of purified rD2BPL3 required for agglutination was 12.5 μg/mL (Figure 6A), while that of native BPL3 was 0.78 μg/mL. rD1BPL3 did not show agglutination activity. The hemagglutination activity was clearly inhibited by pre-incubation with *N*-acetyl-d-glucosamine, *N*-acetyl-d-galactosamine, and fetuin, similar to the results obtained for native BPL3 (Figure 6B). The minimum inhibitory concentration of GlcNAc was 62.5 mM and that of GalNAc was 31.3 mM, corresponding to those of the native form of BPL3 (Table 1). rD2BPL3 also showed similar heat-stability to that of native BPL3 and the protein did not require divalent ions to maintain its sugar binding activity (data not shown).

### 2.5. Glycan Micro-Array Analysis

**Figure 7:**
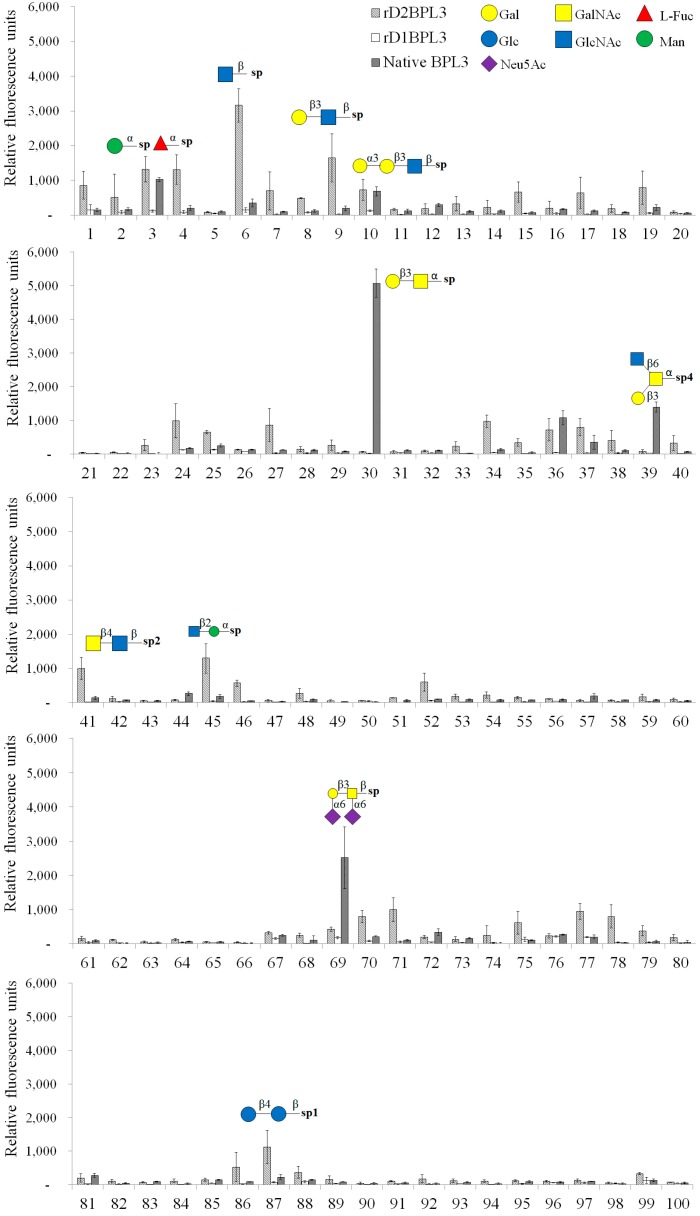
Glycan array of recombinant and native BPL3. Relative fluorescence units were calculated using an array analysis program (RayBioTech). The signal exceeding 1000 units is marked with the glycan structure.

We used a glycoconjugate microarray to determine the glycan binding properties of rD2BPL3, rD1BPL3, and native BPL3. rD1BPL3 did not bind to any sugars on the Glycan-100 array (Figure 7). rD2BPL3 and native BPL3 exhibited partially different glycan binding specificities, except for the monosaccharide α-Man-Sp (over RFU, Relative Fluorescence Units, 1000), Gal-α-1,3-Gal-β-1,3-GlcNAc-β-Sp and Gal-α-1,4-Gal-β-1,3-GlcNAc-β-Sp (Figure 7, Table 2). Interestingly, native BPL3 did not bind to GlcNAc-β-Sp or GalNAc-β-Sp; these mono-saccharides were able to inhibit hemagglutination activity. rD2BPL3 bound to GlcNAc-β-Sp. Recombinant lectin had specificity to the beta (β-) conformation, e.g., Gal-β-1,3-GlcNAc-β-Sp, LacdiNAc (GalNAc-β-1,4-GlcNAc-β-Sp2), and GlcNAc-β-1,2-Man-α-Sp. Native BPL3 exhibited specificity to the alpha (α-) conformation, e.g., Gal-β-1,3-GalNAc-α-Sp (T-antigen, core structure type 3), GlcNAc-β-1,6-(Gal-β-1,3)-GalNAc-α-*O*-Ser-Sp4, and Neu5Ac-α-2,6-Gal-β-1,3-(Neu5Ac-α-2,6)-GalNAc-β-Sp (Figure 7, Table 2).

## 3. Discussion

In the past few decades, various *Bryopsis plumosa* lectins (Bryohealin, BPL2–4) have been purified [12,13,20,21,22], but their biochemical properties are unclear, owing to the lack of a sufficient amount of active recombinant protein for analyses. In particular, the high sequence similarity between BPL3 and BPL4 (60%) and similar molecular properties have limited their applications [12,13]. Thus, recombinant protein production was necessary.

Monomeric (rD1BPL3) and dimeric (rD2BPL3) proteins were designed based on native *BPL3* cDNA and expressed in a bacterial expression system. rD2BPL3 was highly expressed, while the monomeric recombinant showed weak expression or no expression. The bacterial expression system was a sufficient substitute for lectin production from native sources. The expression efficiency of rD2BPL3 was affected by temperature, but IPTG concentration had minimal effects. Approximately 3 mg of rD2BPL3 was produced from 1 L bacterial culture, while the yield of BPL3 was 0.017 mg/g of *Bryopsis plumosa* (data not shown) [12]. Although the productivity was lower than that reported for other recombinant lectins (16.5 mg/L for rRhodobindin [14], 16 mg/L for frutalin [23], and 15 mg/L for *Microcystis viridis* lectin [24]), it is an acceptable value when considering the yield of native BPL3 and other native lectins (0.1 mg/L for *Glycin max* lectin [25], 2–5 mg for *Pisum sativum* lectin [26], and 5 mg/L *Allium sativum* [27]).

Recombinant lectin production is an efficient way to overcome obstacles to the application of lectins derived from natural sources because it guarantees a substantial supply of pure lectins for biomedical applications [28]. Despite their advantages, prokaryotic expression systems have issues with respect to the creation of a proper lectin structure, leading to the frequent production of a biologically inactive protein [14]. Eukaryotic expression systems overcome the limitations of prokaryotic expression systems owing to their post-translational modification ability (e.g., the production of glycosylated proteins). Considering disadvantages with respect to genetic transformation efficiency, the efficiency of expression, immune responses, and the cost of culture [24,29], bacterial expression systems are still widely used for the production of active proteins.

In this study, recombinant BPL3 was successfully produced using an artificially constructed tandem repeat structure in *E. coli*. Although tandem repeat domain structures have been detected in native lectins, such as the Rhamnose-binding lectin from *Silurus asotus* eggs [17], Galectin from *Caenorhabditis elegans* [30], and mannose-binding lectin from *Boodlea coacta* [18], the contribution of tandem repeat structure to recombinant protein production is still unclear.

Previously, Han et al. found that the red algal lectin Rhodobindin consists of a heterologous tandem repeat sequence, and this structure may contribute to protein solubility and hemagglutination activity [14]. The tetrameric structure is advantageous relative to the dimeric form, with 15–30 times greater activity levels for the same concentration [14]. Mixing 2 dimeric domains did not affect their activity; in fact, the activity was enhanced by the formation of the tetrameric structure. Therefore, the construction of a tandem repeat structure is a potential tool for the production of recombinant lectin [14,31]. The expression efficiency of rD2BPL3 was enhanced and hemagglutination activity was 15 times weaker than that of native BPL3. Therefore, we assume that the hemagglutination activity can be improved by construction of trimeric or tetrameric forms, as reported in the previous study [14]. These results support the hypothesis that the tandem repeat structure facilitates lectin production in *E. coli*.

Although an enhancement in lectin solubility was predicted in a previous study, rD2BPL3 could not be solubilized in a common buffer system, PBS. Most lectins contain at least two domains that interact by dimerization or multimerization e.g., [32]. The peptide sequence of BPL3 exhibited similarity to an H type lectin, *Helix pomatia* Agglutinin (HPA), produced by invertebrates [12]. HPA contains two trimeric peptides linked by intramolecular disulfide bonds [33]. Trimerization of HPA occurs by a strong hydrophobic cluster with amino acids in the N- and C-terminal regions of the neighboring monomer (Supplementary Figure S4). rD2BPL3 designed with two identical domains is probably insufficient to generate hydrophobic interactions between domains, and the formation of inclusion bodies resulted. We assumed that enhanced solubility and activity may be possible by constructing a trimeric repeat structure (Supplementary Figure S4). rD2BPL3 was solubilized in denaturing conditions and refolded by flash dilution methods. 

rD2BPL3 showed similar sugar specificity to that of native BPL3. rD1BPL3 was unable to agglutinate human blood cell in this study, but this result needs further validation considering the fact that experiments involving rD1BPL3 could not be replicated satisfactorily because it frequently failed to be expressed. Hemagglutination activity was inhibited by treatment with a complementary sugar, D-GlcNAc or D-GalNAc, used at half the concentrations required to inhibit the activity of rD2BPL3. The concentrations of the complimentary sugars were determined based on the assumption that the affinity of rD2BPL3 for the sugars may be less than that of native BPL3 due to misfolding or the presence of urea in the buffer, which can break ionic bonds and induce a hydrophobic effect [34]. Recombinant acylpeptide hydrolase displayed 15% of its original activity at 8 M urea. It has been reported that protein secondary structure is destroyed at urea concentrations of 2 M and higher [35].

Moreover, biochemical properties, such as the divalent ion requirement and heat stability, were undistinguishable from those of native BPL3. These results can be explained by the similarity in protein structure between rD2BPL3 and native BPL3.

A glycan array is a powerful tool for functional glycomics [36]. We used a glycan array to compare glycan recognition properties between native and recombinant proteins. In contrast to the inhibition test results, the array results indicated that rD2BPL3 and native BPL3 differed with respect to glycan binding properties on the anomeric center of glycan and amino-glycan. Native BPL3 could not bind to β-GlcNAc and β-GalNAc, but the recombinant exhibited strong binding to β-GlcNAc. Native BPL3 showed a preference for the alpha (α-) conformation of amino sugars, and rD2BPL3 exhibited binding to the beta (β-) conformation of amino sugars, such as LacdiNAc. The carbohydrates, α-GalNAc, β-GlcNAc and β-GalNAc, exhibit only slight structural differences. The molecules are designated α- and β- based on the orientation of the hydroxyl group at carbon 1 (C1), while the designations Gal and Glc are based on the orientation of the hydroxyl group at carbon 4 (C4). In nature, these sugars mostly occur as a mixed form of amino sugars (alpha (α-) and beta (β-) conformation). Thus, treatment with these sugars could inhibit the agglutination of human erythrocytes.

The distance between C1 of GlcNAc and candidate binding site amino acids in artificially constructed rD2BPL3—^78^aspartic acid (D78) and ^35^Serine(S35)—was shorter than that between native BPL3 (Supplementary Figure S4). Dimerization likely causes the BPL3 structure to be tighter than that of rD1BPL3 or native BPL3, and this could explain the binding differences between the two forms. The presence of urea in the buffer could be another reason for these differences. The secondary structure of recombinant acylpeptide hydrolase was destroyed in a system containing urea, resulting in loss of enzyme activity [35]. It is difficult to investigate the difference in sugar specificity without structural analysis. Therefore, crystallographic analysis might be needed for deeper understanding of the binding differences. Recombinant and native lectins do not show identical properties. In a comparative analysis, recombinant and native frutalin exhibited different binding properties in prostate tissues [37]. The distinct carbohydrate-binding affinities of the two forms explain this difference in binding [37,38].

Glycosylation and post-translational modifications were not important factors, as evidenced by the lack of post-translational modifications of native BPL3 in a mass spectrometry analysis [12]. The recombinants were consistent with lectins reported in previous studies. In fact, BPL3 was isolated from a mixture of lectins (Bryohealin) based on a competitive binding assay according to sugar specificity (GlcNAc >> GalNAc) [12].

Native BPL3 may consist of 2–6 domains with hydrophobic interactions. BPL3 lacked free sulfhydryl groups; thus, interactions between domains may be flexible. The recombinant protein contained a peptide linker (4 amino acids) to connect homologous domains, resulting in potential tension in the structure. Recombinant WFA with a C272 mutation showed limited binding specificity to GalNAc-terminated glycans [39]; thus, the cysteine residue is important for the maintenance of activity. BPL3 had two intramolecularly connected cysteines. Using the bacterial recombinant system, disulfide bond formation was difficult. It could be concluded that the formation of disulfide bonds is an important determinant of sugar specificity.

The purity of native BPL3 was not high, and a BPL4 band was detected in the SDS-PAGE results (Supplementary Figure S3). In addition, sequences encoding more than four peptides similar to BPL3 were found in the *Bryopsis* genome (unpublished, data not shown, cut-off value: e-50). A mixture of lectins could interact mutually, resulting in structural changes that may influence carbohydrate specificity. The effect of denaturants on protein structure is another candidate hypothesis. The denaturant may affect the protein conformation. In a solution with a denaturant, alterations in protein structure and the solvent structure around the protein are possible [40]. However, the precise mechanisms underlying differences in sugar specificity between native and recombinant proteins are unclear; thus, more intensive studies of protein structure are needed.

HPA is predicted to protect fertilized eggs from bacteria and is part of the innate immune system of the snail [33]. Several sea slugs that are closely related to the snail do not have any such lectins, but symbiotic algae, *Bryopsis* spp., produce similar lectins. We predict that *Bryopsis* lectins have two roles, i.e., protection from mechanical damage and from bacteria in the vicinity of damaged cells and protection of fertilized eggs of symbiotic sea slugs from bacteria; protoplast formation via mechanical damage by sea slugs is a reproduction strategy [41]. BPL3 has potential as an anti-microbial reagent.

Diverse applications of lectins or sugar-binding proteins have been reported [11,42]. High mannose-binding lectin has received attention owing to its potential to inhibit HIV-1 and influenza virus [18]. Lectins have been identified as simple and convenient for histochemical analyses or as biomarkers for disease detection. HPA (with specificity to α-GalNAc, α-GlcNAc, and α-Gal) has been studied in the normal human prostate, benign prostatic hyperplasia, and prostatic carcinoma [43]. WFA has been used as a tool for revealing areal borders and subdivisions. As LacdiNAc (β-d-GalNAc-[1→4]-d-GlcNAc) is associated with tumor malignancy in leukemia, prostate, pancreatic, ovarian, and liver cancers, WFA is promising for cancer glycobiomarker detection [10,39]. rD2BPL3 recognizes LacdiNAc, similarly to WFA; thus, rD2BPL3 is strong candidate for the development of a cancer glycobiomarker.

In our study, recombinant BPL3 was successfully produced using an artificially constructed tandem repeat structure and it demonstrated various advantages for the preparation of pure lectin for industrial purposes. This method may be useful for the production of active proteins. Both lectins (native and recombinant) could be useful histochemical biomarkers.

## 4. Materials and Methods

### 4.1. Preparation of Native BPL3

Native BPL3 was prepared according to the methods of Han et al. [12]. *Bryopsis plumosa* was ground to a fine powder in liquid nitrogen and dissolved in 5 volumes of 1× phosphate-buffered saline (PBS). Cell debris was removed by centrifugation and the supernatant was collected as a crude extract. The supernatant was directly evaluated by GalNAc-agarose chromatography and BPL3 was eluted by adding 0.2 M GlcNAc in 1× PBS. The purified protein was confirmed by SDS-PAGE. Fractions with active proteins were pooled and dialyzed in 1× PBS overnight.

### 4.2. Cloning and Construction of the Recombinant Protein

The *BPL3* cDNA sequence was obtained from the NCBI database (Accession number KX867966). The cDNA was codon-optimized to bacteria K-12 using Geneious ver. 8.1. cDNA was synthesized to mimic the protein with a two-homologous domain fusion structure (rD2BPL3) with restriction enzyme sites from Bioneer (Deajeon, Korea) and cloned into the pBHA vector. *BPL3* DNA was digested and cloned into pET28a (+) (Invitrogen, Carlsbad, CA, USA) (Figure 1). The synthesized *BPL3* and pET28a (+) were digested with two enzymes, *BamH*I and *Sac*I (monomeric, rD1BPL3) or *BamH*I and *Hind*III (dimeric, rD2BPL3), at 37 °C for 2 h. After digestion, the DNA was purified using the Qiagen Gel Extraction Kit (Valencia, CA, USA). The purified DNA was cloned into pET28a (+) by incubation at 12 °C overnight with 4 units of T4 DNA ligase. The plasmid was transformed into the cloning host DH5α and spread on LB agar plates containing 25 μg/mL kanamycin. The positive colonies were isolated and sub-cultured in 10 mL of LB-kanamycin medium. The plasmid containing the *BPL3* sequence was purified and stored at −20 °C until use.

*pET28a::BPL3* was transformed into the expression hosts BL21(λDE3), BL21(DE3)*pLys*S, BL21(DE3) codon-plus RIL, and Rosetta(DE3) (Invitrogen, Carlsbad, CA, USA) to determine the optimal host system. The transformants were spread on LB agar plates containing 25 μg/mL kanamycin. The positive colonies were isolated and sub-cultured in 10 mL of LB-kanamycin medium.

### 4.3. Optimization of rBPL3 Expression 

The transformants were inoculated in LB medium containing kanamycin (25 μg/mL) and cultured overnight at 37 °C. The subculture was diluted 1:100 in 100 mL of LB medium and grown for 1–4 h at 37 °C in an Erlenmeyer flask in a shaker until reaching OD 0.4–0.6. When the designated OD was reached, 1 mL of sample was removed from the flask and the cell pellet was collected by centrifugation (un-induced control). To induce the protein, IPTG (final concentration, 0.4 mM) was added and cultured at various temperatures (20 °C, 25 °C, 30 °C, and 37 °C) overnight. To determine the optimum concentration of IPTG, various concentrations of IPTG (0.1, 0.2, 0.4, and 1 mM) were added to the bacterial culture (OD 0.4–0.6) overnight. The effects of various incubation times (1, 3, and 5 h and overnight) were determined by incubating bacteria after the addition of IPTG (0.4 mM). Aliquots (5 mL) were collected after incubation in different conditions.

Total proteins from each cell culture were extracted and analyzed to determine the expression levels of rD1BPL3 and rD2BPL3. Total protein extracts were prepared by heating samples for 5 min at 90 °C after treatment in 1× SDS-PAGE sample buffer (0.2 mL/mL culture) to the cell precipitates. The extracts were centrifuged at 20,000× *g* and the supernatants were used directly for SDS-PAGE. Soluble fractions were obtained from cell precipitates after cell culture (5 mL). Resuspended samples in 1 mL of extraction buffer (1× PBS, 10 mM imidazole, 1 mM PMSF, pH 7.2) were sonicated at a 15% amplitude, repeated 20 times, with 3-second on/off periods. Supernatants were collected by centrifugation at 20,000× *g* for 10 min. The expression efficiency was determined by calculating the target band intensity after SDS-PAGE using GelAnalyzer 2010 (http://www.gelanalyzer.com/). 4.4. Purification of rBPL3.

### 4.4. Purification of rBPL3.

The bacterial cell culture (500 mL) was centrifuged at 5000× *g* for 10 min and resuspended in 50 mL of urea extraction buffer (50 mM NaH_2_PO_4_, 300 mM NaCl, 8 M urea, pH8.0). Suspensions were sonicated at a 15% amplitude, repeated 20 times with 3-second on/off periods, and centrifuged at 20,000× *g* for 10 min to pellet the cellular debris. The supernatant was collected as a crude extract. The bacterial extract (50 mL) was directly added to 1 mL Ni-NTA chromatography columns (Qiagen) using the FPLC chromatography system (Bio-Rad, Richmond, CA, USA) with a 1 mL/min flow-rate. The column was washed with 15 volumes of wash buffer, 50 mM NaH_2_PO_4_, 300 mM NaCl, 8 M urea, 25 mM imidazole, pH 8.0. Recombinant BPL3 was eluted with an imidazole step gradient, 5 volumes of 75 mM, 125 mM, and 250 mM imidazole in extraction buffer. Fractions were collected and analyzed by SDS-PAGE. Fractions including the pure protein were pooled.

### 4.5. Refolding of rBPL3 

rD2BPL3 inclusion bodies were refolded by the flash dilution method [44]. Purified denatured rD2BPL3 was filtered using a 0.45-µm syringe filter and quickly added to 2, 4, 8, 16, and 40 volumes of refolding buffer (1× PBS with 0.3 M NaCl, pH 7.5). The final concentration of protein was approximately 5–100 µg/mL. The diluted sample was incubated at 20 °C for 3 h. The insoluble material was removed by centrifugation at 20,000× *g* for 10 min at room temperature and the supernatant was collected as refolded rD2BPL3. For quantifying the soluble fractions, diluted samples were re-concentrated to original volume using speed-vac evaporator (Labcono, MO, USA). Same volume of sample was subjected to SDS-PAGE, and the soluble fraction was quantified based on protein band intensity.

### 4.6. Hemagglutination Activity Assay 

Hemagglutination activity was tested following the protocols described by Han et al. [12]. Human blood type B was obtained from a healthy donor and washed with PBS. A serial two-fold dilution of the purified rD2BPL3 was made in a final volume of 25 µL of PBS in 96-well microtiter plates, and 25 µL of erythrocyte suspension (4%) was added to each well. The minimum amount of lectin needed for agglutination was defined as 1 hemagglutinating unit (HU).

### 4.7. Determination of Carbohydrate Specificity

Carbohydrate specificity was determined by inhibition test of hemagglutination activity and a glycan micro-array.

For the inhibition test, mono- and disaccharides at 500 mM or 100 mg/mL glycoprotein were used as inhibitors of lectin: *N*-acetyl-glucosamine, *N*-acetyl-galactosamine, l-fucose, d-galactose, d-glucose, d-mannose, d-fructose, β-lactose, d-maltose, and fetuin. Serial two-fold dilutions of sugar samples were prepared in PBS and mixed with an equal volume of 4 HU rD2BPL3 and native BPL3. An equal volume (25 µL) of a 4% human erythrocyte suspension was added to the sugar-lectin mixture. The minimum inhibitory concentration of the sugar in the final reaction mixture was calculated.

### 4.8. Glycan Microarray

A glycan microarray analysis was performed by Ebiogen (Seoul, Korea). The Glycan Array kit was purchased from RayBioTech (Norcross, GA, USA). An array containing 100 synthetic glycans printed in quadruplicate on a glass slide was used. Label-based detection was performed according to the manufacturer’s protocols. Biotinylated recombinant lectins and native lectins at 50 μg/mL were added to array wells and incubated for >3 h with gentle rocking. The glass slide was washed with 1× wash buffer I and II, provided in the kit. Glycan-lectin binding was detected by incubation with Cy3 equivalent dye-conjugated streptavidin for 1 h at room temperature. For cyanine-3 detection, the signals were visualized using a microarray laser scanner (Genfix 4100A; Molecular Devices, Sunnyvale, CA, USA) with excitation at 554 nm and emission at 568 nm. Data extraction was performed using the microarray analysis software Genfix. Glycan array data were normalized and analyzed using RayBio Analysis software (GA-Glycan-100-SW, RayBioTech).

### 4.9. Effect of Temperature and Divalent Cations on the Agglutination Activity

Heat stability was examined according to the methods of Han et al. [13]. Heated aliquots of purified rD2BPL3 were prepared by incubation at various temperatures (4–100 °C) for 30 min. The samples were cooled to room temperature and insoluble materials were removed by centrifugation at 12,000× *g* for 1 min. The results were expressed as the relative hemagglutination activity shown by the heated samples compared to the non-heated sample (control), representing 100%. The effect of divalent metal ions was determined by adding EDTA or CaCl_2_ at 5 mM in the protein solution.

### 4.10. Mass Spectrometry

Protein bands obtained by SDS-PAGE were excised, in-gel digested with trypsin, and cleaned with Zip-Tip (Millipore, Billerica, MA, USA). Mass analyses were performed using Capillary LC-Nano ESI-MS with a 6545 Q-TOF LC/MS (Agilent Technologies, Santa Clara, CA, USA). Samples were applied to a ZORBAX 300SB-C8 column (1 × 50 mm, 3.5 μm; Agilent) equilibrated with 0.1% (*v*/*v*) formic acid in mass grade water and eluted by a gradient between water and 100% acetonitrile at a flow rate of 10 µL/min. The tuning parameters used for mass analyses were as follows: capillary temperature 300 °C, source voltage 1.9 kV, skimmer voltage 45 V, and fragmentor voltage 175 V.

## 5. Conclusions

Recombinant BPL3 from the coenocytic marine green alga *B. plumosa* was developed using an *E. coli* expression system combined with an artificially constructed tandem repeat structure. The repeat domain contributed to the high expression of the active protein. The recombinant protein recognized *N*-acetyl-β-d-glucosamine, Gal-β-1,3-GlcNAc, LacdiNAc, and GlcNAc-β-1,2-Man. The process developed in this study was suitable for the quality-controlled production of high amounts of soluble recombinant lectins. These results indicate that both lectins (native and recombinant) may have applications as histochemical biomarkers for cancer.

## Figures and Tables

**Table 1:** Inhibition of hemagglutination activity of native BPL3 and rD2BPL3 by various substances.

Substance	Minimum Inhibitory Concentration	
	Native BPL3	rD2BPL3
Fetuin	19.53 ^§^	4.88 ^§^
d-Mannose	NI	NI
l-Fucose	NI	NI
d-Fructose	NI	NI
β-Lactose	NI	NI
*N*-acetyl-d-glucosamine	125	62.5
*N*-acetyl-d-galactosamine	62.5	31.25
d-Galactose	NI	NI
d-Glucose	NI	NI
d-Maltose	NI	NI

^§^ Concentration, μg/mL, NI, the absence of inhibition at 500 mM.

**Table 2:** Overview of carbohydrate structures recognized by rD2BPL3 and native BPL3.

No.	Glycan Structure	RFU (Normalized)	
		rD2BPL3	Native BPL3
**Monosaccharides**			
3	α-Man-Sp	1322	1025
4	α-Fuc-Sp	1313	205
6	β-GlcNAc-Sp	3164	348
**Disaccharides**			
9	Gal-β-1,3-GlcNAc-β-Sp	1646	201
30	Gal-β-1,3-GalNAc-α-Sp	64	5075
41	GalNAc-β-1,4-GlcNAc-β-Sp2	993	134
45	GlcNAc-β-1,2-Man-α-Sp	1290	173
87	d-Cellose-β-Sp1	1119	225
**Gangliosides and Sialylated Oligosaccharides**			
24	Neu5Ac-α-2,6-Gal-β-1,4-Glc-β-Sp	991	178
69	Neu5Ac-α-2,6-Gal-β-1,3-(Neu5Ac-α-2,6)-GalNAc-β-Sp	421	2518
71	Neu5Ac-α-2,6-(Neu5Ac-α-2,3)-Gal-β-1,3-GalNAc-β-Sp	993	96
**Blood Groups, Lewis Antigens and Fucosylated Oligosaccharides**			
34	Neu5Ac-α-2,3-Gal-β-1,3-(Fuc-α-1,4)-GlcNAc-β-[Sialyl Lewis A]-Sp	973	138
**Globo series, Milk Oligosaccharides and GAGs**			
36	Gal-α-1,4-Gal-β-1,3-GlcNAc-β-Sp	721	1082
**O-Glycan, *N*-Glycans and α-Gal**			
39	GlcNAc-β-1,6-(Gal-β-1,3)-GalNAc-α-*O*-Ser-Sp4	77	1398
**Natural Oligosaccharides**			
77	Glc-α-1,6-Glc-α-1,4-Glc-β-Sp1	941	190

## Supplementary Materials

The following are available online at www.mdpi.com/1660-3397/16/1/13/s1, Supplementary Figure S1: Expression efficiency of rD2BPL3 for various expression hosts, Supplementary Figure S2: Confirmation of the peptide sequence using LC-MS/MS, Supplementary Figure S3: Purification of native BPL3, Supplementary Figure S4: Predicted 3-dimensional structure of BPL3, Supplementary Table S1: Glycan array of recombinant and native BPL3. Binding signals were normalized using a program provided by RayBioTech.
