# Supplementary material for: Functional Expression and Characterization of the Recombinant N-Acetyl-Glucosamine/N-Acetyl-Galactosamine-Specific Marine Algal Lectin BPL3
Source: Mar Drugs. 2018 Jan 5;16(1):13. doi: 10.3390/md16010013 (PMC5793061; doi:10.3390/md16010013)
Supplement: Supplementary file 1 [file marinedrugs-16-00013-s001.pdf]

Supplementary Figure S1.

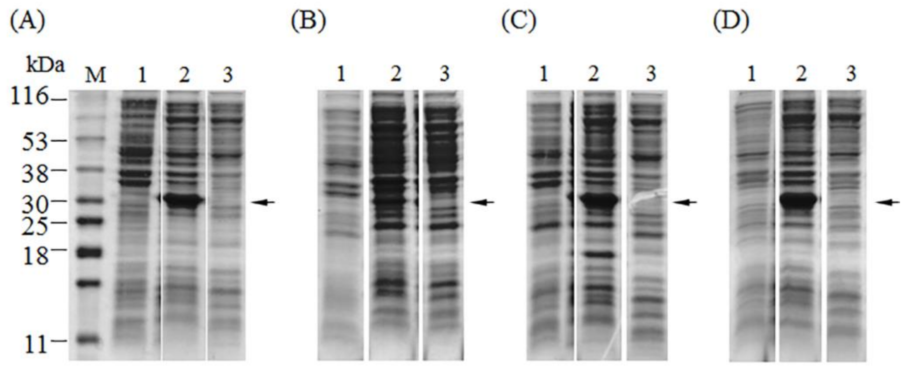

Figure S1. Expression efficiency of rD2BPL3 for various expression hosts. (A) BL21(DE3), (B) BL21(DE3)pLysS, (C) BL21-CodonPlus(DE3)-RIL, (D) Rosetta(DE3). M, Molecular weight marker; Lane 1, un-induced lysate; Lane 2, IPTG induced insoluble fraction; Lane 3, IPTG-induced soluble fraction. Arrows indicate target proteins.

Supplementary Figure S2.

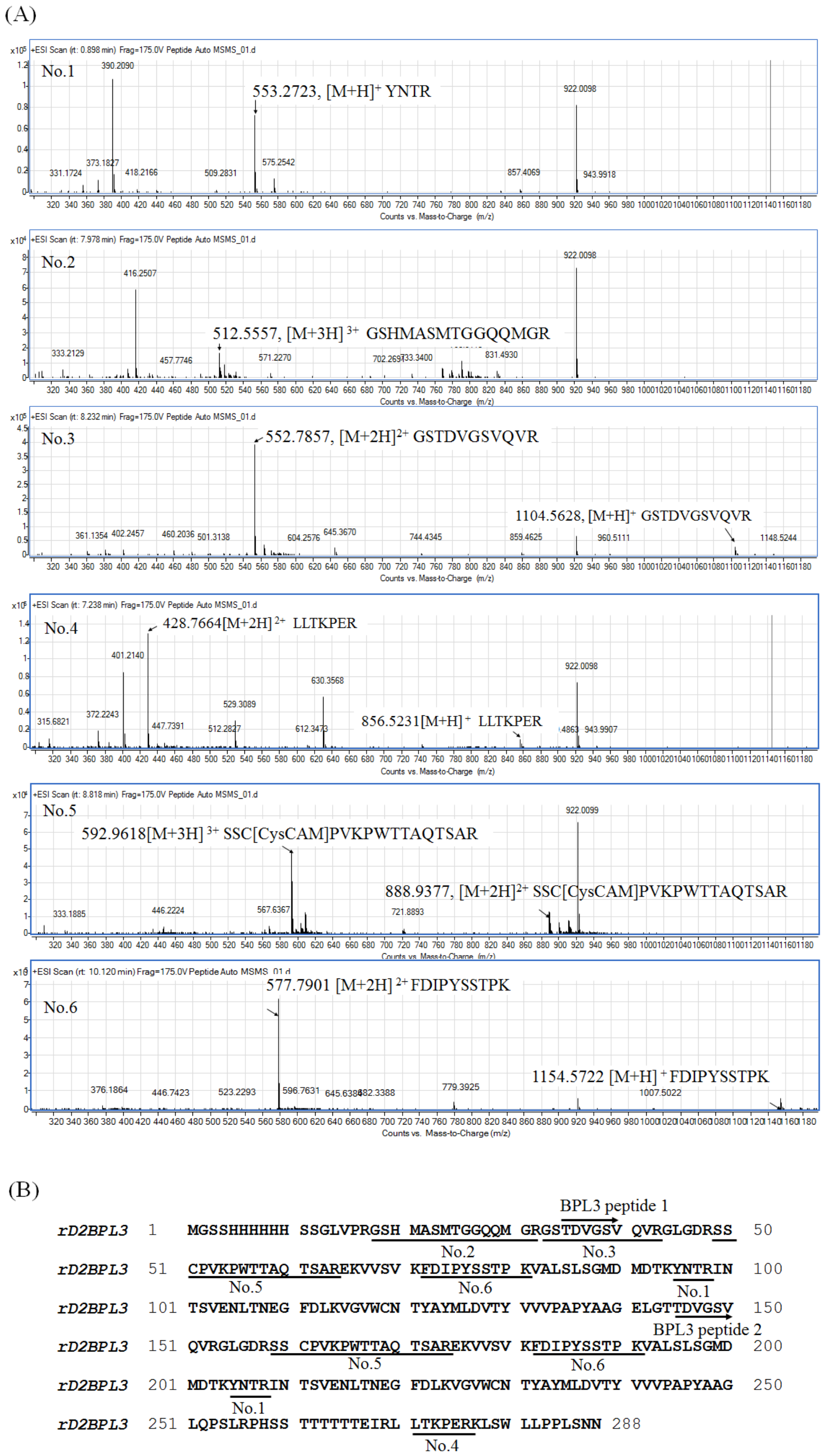

Figure S2. Confirmation of the peptide sequence using LC-MS/MS. (A) Mass spectra of trypsin-digested rD2BPL3; (B) Peptide sequence and matching with mass spectrum data.

Supplementary Figure S3.

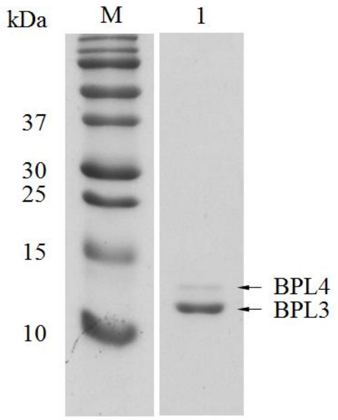

Figure S3. Purification of native BPL3. M, Molecular weight marker; Lane 1, Purified BPL3. Arrows indicate purified proteins.

Supplementary Figure S4.

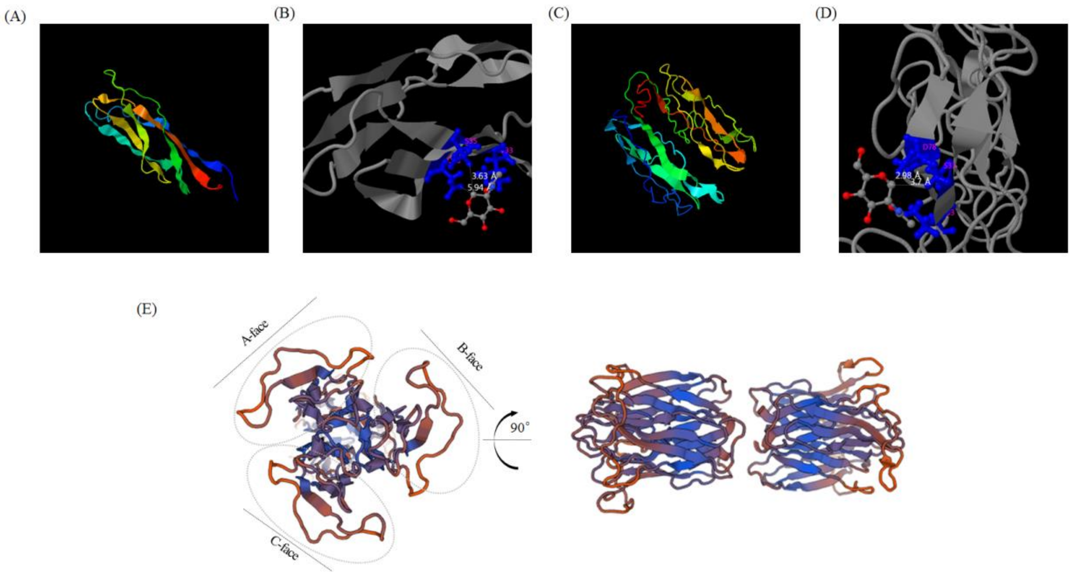

Figure S4. Predicted 3-dimensional structure of BPL3. Structures were analyzed by protein sequence similarity analysis using I-TASSER program (https://zhanglab.ccmb.med.umich.edu/I-TASSER/) and Swiss-Prot 3D structure prediction program (https://swissmodel.expasy.org/) with normal parameters; C-score was typically in the range of [-5, 2], where a C-score of a higher value signifies a model with a higher confidence and vice-versa; (A) native BPL3, c-score was 0.07; (B) enlarged image of native BPL3 binding sites; (C) rD2BPL3, c-score was -3.31; (D) enlarged image for rD2BPL3 binding sites; (E) predicted 3D structure was calculated using Swiss-Model. BPL3 was matched with Helix Pomatia agglutinin (HPA)

Table S1. Glycan array of recombinant and native BPL3. Binding signals were normalized using a program provided by RayBioTech

Substrates	Glycan Structures	RFU (Normalized)	
		rD2BPL3	Native BPL3
**Positive Control**	-	29,419	29,419
**Neg**	-	75	50
**1**	β-Glc-Sp	860±401	148±52
**2**	β-Gal-Sp	510±675	161±44
**3**	α-Man-Sp	1,322±366	1,025±57
**4**	α-Fuc-Sp	1,313±422	205±72
**5**	α-Rha-Sp	86±17	101±25
**6**	β-GlcNAc-Sp	3,164±479	348±112
**7**	β-GalNAc-Sp	698±545	97±15
**8**	Tobramycin	492±13	121±41
**9**	Gal-β-1,3-GlcNAc-β-Sp	1,646±696	201±65
**10**	Gal-α-1,3-Gal-β-1,3-GlcNAc-β-Sp	729±306	685±135
**11**	Neu5Ac-α-2,3-Gal-β-1,3-GlcNAc-β-Sp	165±28	120±55
**12**	Neu5Ac-α-2,6-Gal-β-1,3-GlcNAc-β-Sp	183±143	298±44
**13**	Neu5Gc-α-2,3-Gal-β-1,3-GlcNAc-β-Sp	325±208	108±23
**14**	Neu5Gc-α-2,6-Gal-β-1,3-GlcNAc-β-Sp	229±199	119±33
**15**	Gal-β-1,3-(Fuc-α-1,4)-GlcNAc-β-[Lewis A] –Sp	660±290	70±23
**16**	Gal-β-1,4-Glc-β-Sp	204±198	170±14
**17**	Gal-α-1,3-Gal-β-1,4-Glc-β-Sp	644±450	119±23
**18**	Gal-α-1,4-Gal-β-1,4-Glc-β-Sp	187±106	85±9
**19**	GlcNAc-β-1,3-Gal-β-1,4-Glc-β-Sp	786±480	219±84
**20**	GalNAc-β-1,3-Gal-β-1,4-Glc-β-Sp	87±32	58±19
**21**	Neu5Ac-α-2,3-Gal-β-1,4-Glc-β-Sp	46±13	11±21
**22**	Neu5Ac-α-2,6-Gal-β-1,4-Glc-β-Sp	57±15	7±32
**23**	Neu5Gc-α-2,3-Gal-β-1,4-Glc-β-Sp	266±160	-36±72
**24**	Neu5Ac-α-2,6-Gal-β-1,4-Glc-β-Sp	991±506	178±24
**25**	Gal-β-1,4-(Fuc-α-1,3)-Glc-β-Sp	647±51	255±42
**26**	GalNAc-β-1,3-Gal-α-1,4-Gal-β-1,4-Glc-β-Sp	132±16	130±10
**27**	GlcNAc-β-1,6-GlcNAc-β-Sp	864±482	125±7
**28**	4-P-GlcNAc-β-1,4-Man-β-Sp	148±77	116±23
**29**	Glc-α-1,2-Gal-α-1,3-Glc-α-Sp	260±157	78±13
**30**	Gal-β-1,3-GalNAc-α-Sp	64±13	5,075±429
**31**	Gal-β-1,4-GlcNAc-β-Sp	66±38	105±20
**32**	Gal-β-1,4 -(Fuc-α-1,3)-GlcNAc-β-[Lewis X]–Sp	93±29	104±13
**33**	Neu5Ac-α-2,3-Gal-β-1,4-(Fuc-α-1,3)-GlcNAc-β-[Sialyl Lewis X]-Sp	235±130	25±7
**34**	Neu5Ac-α-2,3-Gal-β-1,3-(Fuc-α-1,4)-GlcNAc-β-[Sialyl Lewis A]-Sp	973±183	138±32
**35**	Neu5Gc-α-2,3-Gal-β-1,3-(Fuc-α-1,4)-GlcNAc-β-[Sialyl Lewis A]-Sp	346±115	45±24
**36**	Gal-α-1,4-Gal-β-1,3-GlcNAc-β-Sp	721±328	1,082±209
**37**	Gal-β-1,4-GlcNAc-β-1,3-Gal-β-1,4-Glc-β- [LNnT]-Sp	797±255	351±207
**38**	GlcA-β-1,4-GlcNAc-α-1,4-GlcA-β-Sp	401±296	104±23
**39**	GlcNAc-β-1,6-(Gal-β-1,3)-GalNAc-α-O-Ser- Sp4	77±48	1,398±148
**40**	Neu5Ac-α-2,3Gal-β-1,4-(6S)GlcNAc-β-Sp	326±222	72±13
**41**	GalNAc-β-1,4-GlcNAc-β-Sp2	993±332	134±45
**42**	Neu5Ac-α-2,8-Neu5Ac-α-2,3-Gal β-1,4-Glc-β-Sp	102±79	65±16
**43**	Neu5Gc-α-2,8-Neu5Ac-α-2,3-Gal-β-1,4-Glc-β-Sp	40±21	46±13
**44**	GalNAc-α-1,3-(Fuc-α-1,2)-Gal-β-1,4-Glc-β- [Blood A antigen tetrose]-Sp1	70±18	256±48

**45**	GlcNAc-β-1,2-Man-α-Sp	1,290±426	173±53
**46**	Neu5Ac-α-2,3-Gal-β-Sp1	565±90	42±4
**47**	Gal-β-1,3 -GalNAc-β-1,3-Gal-β-Sp1	53±34	12±29
**48**	Glc-α-1,2-Gal-α-Sp	264±150	74±28
**49**	Gal-β-1,4-(Fuc-α-1,3)-GlcNAc-β-1,3-Gal-β-Sp1	43±39	2±25
**50**	Neu5Ac-α-2,3-Gal-β-1,4-(Fuc-α-1,3)-Glc-β- [3-Sialyl-3-fucosyllactose/ F-SL]-Sp1	54±9	2±30
**51**	GlcNAc-β-1,4-GlcNAc-β-Sp1	135±3	57±34
**52**	β-D-GlcA-Sp	595±257	99±10
**53**	Gal-β-1,4-(6S)GlcNAc-β-Sp	169±71	84±16
**54**	GlcNAc-α-1,3-(Glc-α-1,2-Glc-α-1,2)-Gal-α-1,3-Glc-α-Sp	214±90	64±24
**55**	Gal-β-1,3-GalNAc-β-1,4-(Neu5Gc-α-2,3)-Gal-β-1,4-Glc-β-Sp1	138±37	69±8
**56**	Sisomicin Sulfate	105±9	80±24
**57**	GalNAc-α-1,3-(Fuc-α-1,2)-Gal-β-[Blood A antigen trisaccharide]-Sp1	59±30	181±73
**58**	Fuc-α-1,2-Gal-β-1,4-GlcNAc-β-[Blood H antigen trisaccharide]-Sp1	57±21	72±5
**59**	Gal-α-1,3-(Fuc-α-1,2)-Gal-β-[Blood B antigen trisaccharide]-Sp1	158±92	72±24
**60**	Fuc-α-1,2-Gal-β-1,3-GlcNAc-β-1,3-Gal-β-1,4-Glc-β-[LNFP I]-Sp1	81±51	44±17
**61**	Fuc-α-1,2-Gal-β-1,4-Glc-β-[Blood H antigen trisaccharide]-Sp1	148±60	84±24
**62**	Gal-α-1,3-(Fuc-α-1,2)-Gal-β-1,4-Glc-β-[Blood B antigen tetrasaccharide]-Sp1	108±20	14±7
**63**	(Fuc-α-1,2)-Gal-β-1,4-(Fuc-α-1,3)-GlcNAc-β- [Lewis Y]-Sp1	49±28	24±20
**64**	(Fuc-α-1,2)-Gal-β-1,3-(Fuc-α-1,4)-GlcNAc-β- [Lewis B]-Sp1	122±31	60±18
**65**	Gal-β-1,3-(Fuc-α-1,4)-GlcNAc-β-1,3-Gal-β-1,4-(Fuc-α-1,4)-Glc-β- [Lewis A]-Sp1	45±10	47±17
**66**	Gal-β-1,3-GalNAc-β-Sp1	39±16	17±9
**67**	Gal-β-1,3-(Neu5Ac-α-2,6)-GalNAc-β-Sp	318±43	240±32
**68**	Neu5Ac-α-2,6-Gal-β-1,3-GalNAc-β-Sp	247±59	101±124
**69**	Neu5Ac-α-2,6-Gal-β-1,3-(Neu5Ac-α-2,6)-GalNAc-β-Sp	421±59	2,518±904
**70**	Neu5Ac-α-2,3-Gal-β-1,3-(Neu5Ac-α-2,6)-GalNAc-β-Sp	789±117	209±12
**71**	Neu5Ac-α-2,6-(Neu5Ac-α-2,3)-Gal-β-1,3-GalNAc-β-Sp	993±349	96±24
**72**	GalNAc-β-1,4-(Neu5Ac-α-2,3)-Gal-β-1,4-Glc-β-[GM2]-Sp	191±44	338±96
**73**	GalNAc-β-1,4-(Neu5Ac-α-2,8-Neu5Ac-α-2,3)-Gal-β-1,4-Glc-β-[GD2]-Sp	131±66	154±22
**74**	Gal-α-1,4-Gal-β-1,4-GlcNAc-β-Sp1	238±289	12±22
**75**	β-D-Rha-Sp	615±331	99±8
**76**	Glc-α-1,4-Glc-β-Sp1	227±59	265±19
**77**	Glc-α-1,6-Glc-α-1,4-Glc-β-Sp1	941±234	190±57
**78**	Maltotriose-β-Sp1	796±340	19±18
**79**	Glc-α-1,6-Glc-α-1,6-Glc-β-Sp1	376±148	62±38
**80**	Maltotetraose-β-Sp1	181±89	43±45
**81**	GlcNAc-α-1,4-GlcA-β-1,4-GlcNAc-α1,4-GlcA-β-Sp	200±124	277±61
**82**	Maltohexaose-β-Sp1	108±54	47±24
**83**	Maltoheptaose-β-Sp1	72±20	90±17
**84**	Acarbose-β-Sp1	113±53	33±14

**85**	D-Pentamannuronic acid-β-Sp1	150±41	146±17
**86**	L-Pentaguluronic acid-β-Sp1	529±439	89±7
**87**	D-Cellose-β-Sp1	1,119±495	225±69
**88**	Gal-α-1,3-Gal-β-Sp1	368±176	141±20
**89**	β-1,4-Xylotetrose-Sp1	155±110	84±9
**90**	Chitin-Trisaccharide-Sp1	45±31	44±28
**91**	KDN-α-2,8-Neu5Ac-α-2,3-Gal-β-1,4-Glc-β-Sp	108±20	59±16
**92**	Neu5Ac-α-2,8-Neu5Gc-α-2,3-Gal-β-1,4-Glc-β-Sp	177±127	34±15
**93**	Neu5Ac-α-2,8-Neu5Ac-α-2,8-Neu5Ac-α-2,3-Gal-β-1,4-Glc-β-Sp3	122±53	72±24
**94**	Neu5Ac-α-2,8-Neu5Ac-α-2,6-Gal-β-1,4-Glc-Sp5	102±42	35±18
**95**	Gal-β-1,3-GalNAc-β-1,4-(Neu5Ac-α-2,3)-Gal-β-1,4-Glc-β-Sp1	115±26	88±28
**96**	Gentamicin Sulfate	104±25	76±19
**97**	Kanamycin Sulfate	128±44	102±6
**98**	Geneticin Disulfate Salt (G418)	61±19	28±23
**99**	Neomycin Trisulfate	333±36	129±37
**100**	SGP	75±6	52±22
